# The Changes in Trends of Lower Gastrointestinal Endoscopy Conducted in Children and Adolescents after the COVID-19 Outbreak in Korea

**DOI:** 10.3390/medicina58101378

**Published:** 2022-10-01

**Authors:** Sang Woo Lee, Ben Kang, Sujin Choi, Byung-Ho Choe, Yu Bin Kim, Kyung Jae Lee, Hyun Jin Kim, Hyo-Jeong Jang, So Yoon Choi, Dae Yong Yi, You Jin Choi, Ju Young Kim, Eun Hye Lee, Yoo Min Lee

**Affiliations:** 1 Department of Pediatrics, School of Medicine, Kyungpook National University, 130 Dongdeok-ro, Jung-gu, Daegu 41944, Korea; 2 Department of Pediatrics, Ajou University School of Medicine, 164, World Cup-ro, Yeongtong-gu, Suwon 16499, Korea; 3 Department of Pediatrics, Hallym University College of Medicine, 1 Hallymdaehak-gil, Chuncheon 24252, Korea; 4 Department of Pediatrics, Chungnam National University Hospital, 282 Munhwa-ro, Jung-gu, Daejeon 35015, Korea; 5 Department of Pediatrics, Dongsan Medical Center, Keimyung University School of Medicine, 1035, Dalgubeol-daero, Dalseo-gu, Daegu 42601, Korea; 6 Department of Pediatrics, Kosin University Gospel Hospital, Kosin University College of Medicine, 262 Gamcheon-ro, Seo-gu, Busan 49267, Korea; 7 Department of Pediatrics, Chung-Ang University Hospital, Chung-Ang University College of Medicine, 102, Heukseok-ro, Dongjak-gu, Seoul 06973, Korea; 8 Department of Pediatrics, Inje University Ilsan Paik Hospital, Juhwa-ro 170, Ilsanseo-gu, Goyang 10380, Korea; 9 Department of Pediatrics, Eulji University Hospital, 95, Dunsanseo-ro, Seo-gu, Daejeon 35233, Korea; 10 Department of Pediatrics, Nowon Eulji Medical Center, Eulji University School of Medicine, 68, Hangeulbiseok-ro, Nowon-gu, Seoul 01830, Korea; 11 Department of Pediatrics, Soonchunhyang University Bucheon Hospital, Soonchunhyang University College of Medicine, 170, Jomaru-ro, Bucheon 14584, Korea

**Keywords:** colitis ulcerative, colonoscopy, COVID-19, Crohn’s disease, inflammatory bowel diseases

## Abstract

*Background and Objectives:* The coronavirus disease 2019 (COVID-19) pandemic has affected medical practice in diverse ways. We aimed to investigate the change in trends of lower gastrointestinal (LGI) endoscopy conducted in children and adolescents after the COVID-19 outbreak in Korea. *Material and Methods:* This was a multicenter, retrospective study conducted in Korea. We included children and adolescents aged <19 years who had undergone their first LGI endoscopy between 2016 and 2020. We compared clinicodemographic and endoscopic factors between groups divided according to the pre- and postCOVID-19 era in Korea. *Results:* We included 1307 patients in this study. Colonoscopies, instead of sigmoidoscopies, were conducted in most patients in the postCOVID-19 era compared to those in the preCOVID-19 era (86.9% vs. 78.5%, *p* = 0.007). The diagnosis of inflammatory bowel disease (IBD) was also significantly higher in the postCOVID-19 era compared to the preCOVID-19 era (47.2% vs. 28.5%, *p* < 0.001). According to multivariate logistic regression analysis, age at LGI endoscopy, LGI bleeding indication, and IBD diagnosis were independently associated with the use of a colonoscopy over a sigmoidoscopy (odds ratio (OR) 1.19, 95% confidence interval (CI) 1.12–1.27, *p* < 0.001; OR 0.56, 95% CI 0.37–0.83, *p* = 0.005; OR 1.80, 95% CI 1.20–2.77, *p* = 0.006, respectively). *Conclusions*: The COVID-19 pandemic has changed LGI endoscopy practice trends of pediatric gastroenterologists in Korea, who tended to perform lesser LGI endoscopies compared to previous years while conducting significantly more colonoscopies than sigmoidoscopies in the postCOVID-19 era. Furthermore, these colonoscopies were significantly associated with the diagnosis of IBD, as well as a significant increase in IBD diagnosis in the postCOVID-19 era.

## 1. Introduction

The coronavirus disease 2019 (COVID-19) pandemic has changed medical practice in many ways. Health care workers are at increased risk of infection and proper safety precautions are required, especially in those who work in the endoscopy unit [1]. Upper gastrointestinal (GI) tract procedures are aerosol-generating and may give rise to the aerosolization of viral particles. Although this has not yet been elucidated in lower GI (LGI) tract procedures, there are still concerns of virus shedding through the feces in asymptomatic patients with COVID-19.

The use of pediatric LGI endoscopy has increased worldwide, probably due to the increasing number of procedures and increasing incidence and recognition of LGI tract diseases, such as inflammatory bowel disease (IBD) [2,3,4]. However, with the spread of the COVID-19 pandemic, changes in the trend or type of LGI performances may have occurred.

For example, endoscopic procedures were indicated in urgent situations and care for patients with cancer during a certain time when the COVID-19 outbreak initially explosively increased in the city of Daegu [5]. During this time, nonurgent procedures were rescheduled to a later date. Thus, pediatric LGI endoscopies were reserved and prioritized for those with absolute indications, even though most centers in South Korea had not regularly shut down after the COVID-19 pandemic.

To the best of our knowledge, no study has been performed on changes in trends of LGI endoscopy conducted in children and adolescents after the COVID-19 pandemic. Therefore, we aimed to investigate this in Korean pediatric patients and evaluate the impact of the COVID-19 outbreak on medical practice in the field of pediatric LGI endoscopy.

## 2. Methods

### 2.1. Patients and Study Design

We conducted a multicenter, retrospective study in 12 medical centers in South Korea: Kyungpook National University Children’s Hospital affiliated with Kyungpook National University Chilgok Hospital, Soonchunhyang University Bucheon Hospital, Ajou University Medical Center, Hallym University Sacred Heart Hospital, Chungnam National University Hospital, Keimyung University Dongsan Hospital, Kyungpook National University Hospital, Kosin University Gospel Hospital, Chung-Ang University Hospital, Inje University Ilsan Paik Hospital, Eulji University Hospital, and Nowon Eulji Medical Center. We included children and adolescents aged <19 years who had benefited from their first LGI endoscopy between January 2016 and December 2020. The study period was set based on the first confirmed case in South Korea, which was reported on 21 January 2020 [6].

We reviewed their electronic medical charts and investigated factors, including age, sex, date of the first LGI endoscopy, endoscopy type, sedation type, indications for LGI endoscopy, diagnosis, and complications. As nodular lymphoid hyperplasia (NLH) is frequently observed in normal children and adolescents, those with mere endoscopic NLH without a clinical diagnosis were considered normal. Patients who had received their first LGI endoscopy between 2016 and 2019 were placed in the preCOVID-19 group, while those who had received their first LGI endoscopy in 2020 were placed in the postCOVID-19 group. Comparative analysis was conducted between these two groups.

### 2.2. Statistical Analysis

Student’s *t*-test or Wilcoxon’s rank-sum test was used for statistical comparison between groups for continuous variables, and a chi-square test or Fisher’s exact test was used for categorical variables. Comparative data for continuous variables are reported as median (interquartile range (IQR)) or mean standard deviation. Univariate and multivariate logistic regression analyses were conducted to examine factors associated with the conductance of a colonoscopy over a sigmoidoscopy. Univariate logistic regression analysis was performed to investigate the crude odds ratio (OR) for each factor. Factors showing a statistical significance (*p* < 0.1) were included in the multivariate logistic analysis. The results were expressed as adjusted ORs with 95% confidence intervals (CIs) and *p* value of less than 0.05 was considered statistically significant. Statistical analyses were conducted using R version 3.2.3 (The R Foundation for Statistical Computing, Vienna, Austria; https://www.r-project.org (accessed on 10 March 2022 ).

### 2.3. Ethics Statement

This study was approved by the Institutional Review Board (IRB) of the Kyungpook National University Chilgok Hospital and all other participating centers, and informed consent was waived due to the retrospective nature of this study (IRB No. 2020-08-007, approved on 14 August 2020). We conducted this study in compliance with the principles of the Declaration of Helsinki.

## 3. Results

### 3.1. Baseline Characteristics of the Patients

A total of 1307 patients had undergone their first LGI endoscopy between January 2016 and December 2020.

A total of 805 (61.6%) were males, and the mean age at diagnosis was 15.0 (IQR 11.0–17.0) years.

Most (79.6%) indications for LGI endoscopy were colonoscopies, and the intubation rate up to the terminal ileum was 93.4% (972/1041). Sigmoidoscopy comprised 20.4% of the LGI endoscopy, and 66.2% of the patients had been intubated distal to the sigmoid colon (176/266). Intravenous sedation was performed in 1235 patients (94.5%), while general anesthesia was given in 8 patients (0.6%) (Table 1).

The most common indication for LGI endoscopy was GI bleeding (37.8%), followed by chronic diarrhea (35.4%), abdominal pain (20.0%), and others (6.8%) (Table 2). A definite diagnosis was made in 828 patients (63.4%). Crohn’s disease (CD) was most commonly diagnosed (21.6%), followed by nonspecific colitis (15.9%), ulcerative colitis (9.7%), and others (16.2%) (Table 3).

One case of bowel perforation was noted during a diagnostic colonoscopy in a patient with suspected CD (0.1%). Four patients had to stop colonoscopy due to irritability associated with the intravenous sedation drug (0.3%).

### 3.2. Comparison of Characteristics between the Pre- and PostCOVID-19 Era

**Figure 1:**
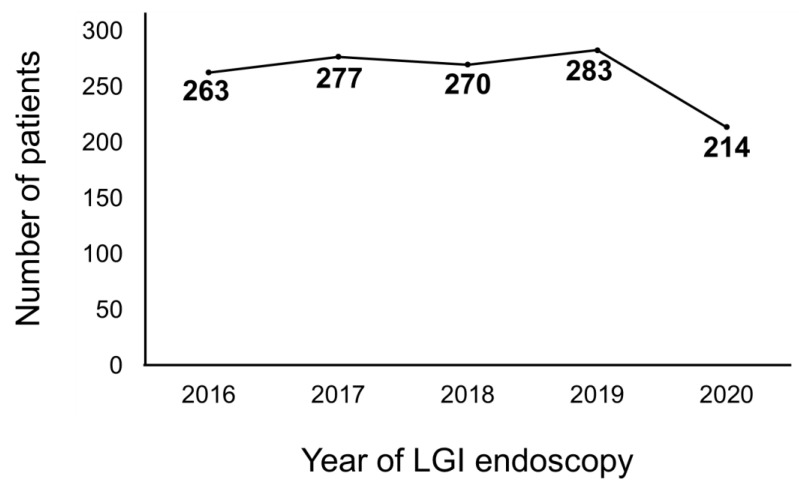
Number of patients that had received a lower gastrointestinal (LGI) endoscopy from 2016 to 2020.

**Figure 2:**
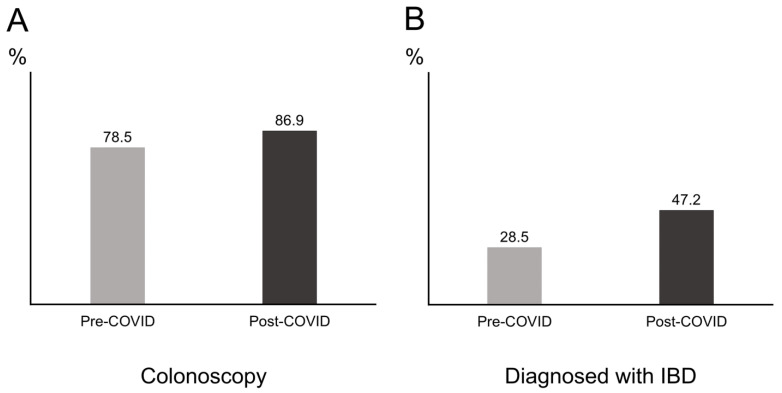
Proportion of patients between groups divided according to the pre- and post-coronavirus infectious disease 2019 (COVID-19) era. (**A**) Proportion of patients receiving a colonoscopy. (**B**) Proportion of patients diagnosed with inflammatory bowel disease (IBD).

A total of 1093 had received an LGI endoscopy between 2016 and 2019, which corresponded to an average of 273 patients per year. Meanwhile, 214 patients benefited from an LGI endoscopy in 2020 (Figure 1). Comparison between the pre- and postCOVID-19 era revealed significant differences in the type of LGI endoscopy and diagnosis. Patients performed more colonoscopies than sigmoidoscopies in the postCOVID-19 era compared to the preCOVID-19 era (86.9% vs. 78.5%, *p* = 0.007) (Figure 2A). IBD was diagnosed significantly more in the postCOVID-19 era compared to the preCOVID-19 era (47.2% vs. 28.5%, *p* < 0.001) (Figure 2B). Comparison of indications between the pre- and postCOVID-19 era revealed relatively higher proportion of indications in accordance to LGI endoscopy guidelines in the postCOVID-19 era compared to the pre-COVID-19 era, although a statistical significance was not observed (Table 4).

### 3.3. Comparison of Characteristics between Patients Receiving Colonoscopies and Sigmoidoscopies

Comparing patients who had received a colonoscopy to those who received sigmoidoscopy, the age of the LGI endoscopy was significantly higher in patients who had received a colonoscopy (median 15.2 vs. 7.5 years, *p* < 0.001). Moreover, diarrhea was the main indication in most patients who had received a colonoscopy (40.1% vs. 17.3%, *p* < 0.001), while patients who had received a sigmoidoscopy had a higher proportion of patients with LGI bleeding as the main indication of the exam (63.2% vs. 31.3%, *p* < 0.001). The proportion of patients receiving their LGI endoscopy without sedation or anesthesia was significantly higher in patients who had received a sigmoidoscopy (23.7% vs. 0.1%, *p* < 0.001). Patients who had received a colonoscopy were likely to have benefited from another colonoscopy in the future than those who had received a sigmoidoscopy (19.9% vs. 12.8%, *p* = 0.01) (Table 5).

### 3.4. Factors Associated with Conducting a Colonoscopy over a Sigmoidoscopy

According to the univariate logistic regression analysis, age at LGI endoscopy, weight at LGI endoscopy, LGI performance year, LGI bleeding indication, chronic diarrhea indication, and IBD diagnosis were each significantly associated with the conductance of a colonoscopy over a sigmoidoscopy. However, according to multivariate logistic regression analysis, age at LGI endoscopy, LGI bleeding indication, IBD diagnosis were independent factors associated with the performance of a colonoscopy over a sigmoidoscopy (age at LGI endoscopy: OR 1.19, 95% CI 1.12–1.27, *p* < 0.001; LGI bleeding indication: OR 0.56, 95% CI 0.37–0.83, *p* = 0.005; IBD diagnosis: OR 1.80, 95% CI 1.20–2.77, *p* = 0.006, respectively) (Table 6).

## 4. Discussion

This study is the first to investigate the changes in trends of LGI endoscopy in children and adolescents after the COVID-19 pandemic. We found that LGI endoscopies were less frequently conducted in the postCOVID-19 era compared to the preCOVID-19 era. However, the number of patients who received a colonoscopy increased in the postCOVID-19 era. Furthermore, the proportion of patients who were diagnosed with IBD had significantly increased in the postCOVID-19 era. Colonoscopies were also significantly associated with the diagnosis of IBD, which indicated a significant increase in IBD diagnosis in the postCOVID-19 era.

Pediatric endoscopic procedures are considered to be a high risk for COVID-19 transmission. Hence, endoscopic care is rapidly changing in the COVID-19 era. Recently, the North American Society of Gastroenterology Hepatology and Nutrition (NASPGHAN) endoscopy and procedures committee proposed guidelines to protect endoscopists from COVID-19 infection [1]. According to the guidelines, endoscopic procedures are recommended in emergent situations, such as life-threatening GI bleeding or bowel obstruction, while the risks and benefits should be weighed in urgent situations. Meanwhile, endoscopic procedures may be postponed in elective situations, such as polypectomy in patients with polyposis. Although the COVID-19 pandemic was relatively well controlled socially in South Korea, and most centers did not have to shut down or limit the use of GI endoscopies, caution is required during the exams. The decrease in sigmoidoscopies is likely the result of reluctance in performing the exams in those with minor symptoms, while the increase in full colonoscopies may have increased with the increase in IBD incidence. In other words, those who definitely require a lower GI endoscopy were likely to have actually received the exam. Moreover, this may be due to greater adherence to LGI guidelines in the postCOVID-19 era. Although statistical significance was not observed, a higher proportion of children and adolescents received a LGI endoscopy in accordance with LGI endoscopy guidelines in the postCOVID-19 era compared to the pre-COVID-19 era.

A colonoscopy is an important tool for the diagnosis and management of digestive disease in children [7]. Pediatric endoscopy has progressed during the past three decades in aspects of diagnosis and treatment. In the last decade, pronounced progress has been observed due to the ongoing development of endoscopy technology and the increase in the number of pediatric endoscopy specialists [8]. To date, there are only a few studies that have investigated the incidence and usefulness of diagnostic or therapeutic endoscopy [7,9]. A few studies were conducted abroad and one domestic study was reported from a single center [2,10,11].

Similar to our findings, the most common primary indications for LGI are LGI bleeding, abdominal pain/bloating symptoms, diarrhea, and IBD [12]. Positive findings in this study were seen in 53.8% (558/1037) of the exams, which is similar to previous results of 45.8% to 70.5% obtained from single-center studies conducted in Korea, Hong Kong, and China [3,11,13]. Previous studies conducted approximately a decade ago have shown that colorectal polyps are most commonly diagnosed during LGI endoscopy in children [3]. Similar to our findings, more recent studies have revealed that IBD is most commonly diagnosed [8]. The incidence of IBD is rapidly increasing in Korea [14], and colonoscopy plays a crucial role in the diagnosis, treatment, follow-up, and monitoring of IBD. Thus, the frequency of colonoscopy exams did not decrease even in the COVID-19 era due to the importance of colonoscopy in discriminating between IBD and noninflammatory causes of chronic diarrhea.

According to a previous study on children, the frequency of conducting a colonoscopy as an initial diagnostic exam for the etiology of colitis-type symptoms was 64% [15]. The decision of whether to conduct a colonoscopy or a sigmoidoscopy as the initial exam in patients with chronic diarrhea is likely to be based on factors such as age, suspected disease, and compliance with bowel preparation. The degree of bowel preparation is a core factor for the success of a colonoscopy, as it is closely related to the quality of the procedure. Patients often consider bowel preparation as the most unpleasant part of the exam [16]. These factors, along with the increased incidence of IBD, may have played a role in the relative increase in the frequency of colonoscopies.

There are yet limited data regarding the change in IBD incidence in the COVID-19 era. However, one study from the Daegu-Kyungpook province of Korea has reported a significant increase in pediatric IBD incidence, especially CD, after the COVID-19 pandemic compared to that before [17]. The authors in that study discussed that the increase in incidence may be attributable to several factors. First, the increase may be merely on the same line of a continuous increase in pediatric IBD in Korea. Second, the relative increase in pediatric IBD incidence may be a result of a sharp decrease in the number of children in the region. Third, the change in lifestyle in the post-COVID-19 era may have influenced this increase. Further data from other regions and countries worldwide are required to elucidate whether this trend of increase seen in pediatric IBD incidence is a worldwide trend or just a regional phenomenon.

This study has some limitations. Caution is therefore required in interpreting the results of this study. Firstly, this study is retrospective. Secondly, the number of patients included in this study was relatively small. Thirdly, this was a multicenter study conducted in some centers in Korea. Therefore, the results may not represent the whole country. Further large-scale multicenter studies based on a nationwide status are required.

## 5. Conclusions

In conclusion, the COVID-19 pandemic has changed LGI endoscopy practice trends of pediatric gastroenterologists in Korea, who tended to perform fewer LGI endoscopies compared to in previous years while conducting significantly more colonoscopies than sigmoidoscopies in the postCOVID-19 era. Furthermore, these colonoscopies were found to be significantly associated with IBD diagnosis, as well as with a significant increase in IBD diagnosis in the postCOVID-19 era.

## Figures and Tables

**Table 1:** Baseline characteristics.

	*n* (%) (*n* = 1307)
Male	805 (61.6)
Age at LGI endoscopy (years)	15.0 (11.0–17.0)
Height (cm)	160.4 (142.5–170.0)
Weight (kg)	50.8 (34.7–60.2)
BMI (kg/m^2^)	19.0 (16.6–21.8)
Year of first LGI endoscopy	
2016	263 (20.1)
2017	277 (21.2)
2018	270 (20.7)
2019	283 (21.7)
2020	213 (16.3)
LGI endoscopy type	
Colonoscopy	1041 (79.6)
Sigmoidoscopy	266 (20.4)
Sedation/Anesthesia type	
Intravenous sedation	1235 (94.5)
General anesthesia	8 (0.6)
None	64 (4.9)

Values are expressed as number (%) or median (interquartile range). BMI, body mass index; LGI, lower gastrointestinal.

**Table 2:** Indications for lower gastrointestinal endoscopy.

	*n* (%) (*n* = 1307)
Gastrointestinal bleeding	494 (37.8)
Chronic diarrhea	463 (35.4)
Abdominal pain	262 (20.0)
Weight loss	22 (1.7)
Perianal discharge	19 (1.5)
Growth retardation	18 (1.4)
Anemia	13 (1.0)
Foreign body removal	7 (0.5)
Vomiting	4 (0.3)
Lip pigmentation	2 (0.2)
Hypoalbuminemia	1 (0.1)
Fever of unknown origin	1 (0.1)
Oral ulcer	1 (0.1)

**Table 3:** Diagnosis of patients based on clinical and endoscopic findings.

	Colonoscopy (*n* = 1044)	Sigmoidoscopy (*n* = 263)	Total (*n* = 1307)
Normal	402 (38.5)	85 (32.3)	487 (37.3)
Crohn’s disease	273 (26.1)	9 (3.4)	282 (21.6)
Non-specific colitis	134 (12.8)	74 (28.1)	208 (15.9)
Ulcerative colitis	98 (9.4)	29 (11.0)	127 (9.7)
Colorectal polyps	60 (5.7)	18 (6.8)	78 (6.0)
EGID	26 (2.5)	6 (2.3)	32 (2.4)
Pseudomembranous colitis	4 (0.4)	16 (6.1)	20 (1.5)
Hemorrhoids	15 (1.4)	2 (0.8)	17 (1.3)
Allergic colitis	0 (0.0)	12 (4.6)	12 (0.9)
Foreign body	4 (0.4)	3 (1.1)	7 (0.5)
Proctitis	6 (0.6)	1 (0.4)	7 (0.5)
GVHD	1 (0.1)	4 (1.5)	5 (0.4)
Diverticulum/Diverticulitis	4 (0.4)	0 (0.0)	4 (0.3)
Solitary rectal ulcer	3 (0.3)	1 (0.4)	4 (0.3)
Angiodysplasia	3 (0.3)	0 (0.0)	3 (0.2)
Behcet’s disease	3 (0.3)	0 (0.0)	3 (0.2)
Sigmoid volvulus	1 (0.1)	2 (0.8)	3 (0.2)
Terminal ileitis	3 (0.3)	0 (0.0)	3 (0.2)
TB enterocolitis	2 (0.2)	0 (0.0)	2 (0.2)
Collagenous colitis	1 (0.1)	0 (0.0)	1 (0.1)
Ischemic colitis	0 (0.0)	1 (0.4)	1 (0.1)
Microvillous inclusion disease	1 (0.1)	0 (0.0)	1 (0.1)

EGID, eosinophilic gastrointestinal disease; GVHD, graft-vs-host disease; TB, tuberculosis.

**Table 4:** Comparison of indications between patients receiving a LGI endoscopy in the preCOVID-19 era and postCOVID-19 era.

	PreCOVID-19 Era (*n* = 1093)	PostCOVID-19 Era (*n* = 214)	*p*-Value
Indication in accordance to LGI guidelines			0.261
Yes	864 (79.0)	177 (82.7)	
No	229 (21.0)	37 (17.3)	

**Table 5:** Comparison of characteristics between patients receiving colonoscopies and sigmoidoscopies.

	Colonoscopy (*n* = 1041)	Sigmoidoscopy (*n* = 266)	*p*-Value
Male	649 (62.3)	156 (58.6)	0.300
Age at diagnosis (years)	15.2 (12.9–17.1)	7.5 (2.5–15.6)	<0.001
Sedation/Anesthesia type			<0.001
Intravenous sedation	1035 (99.4)	200 (75.2)	
General anesthesia	5 (0.5)	3 (1.1)	
None	1 (0.1)	63 (23.7)	
Indication: LGI bleeding	326 (31.3)	168 (63.2)	<0.001
Indication: chronic diarrhea	417 (40.1)	46 (17.3)	<0.001
Diagnosis: IBD	374 (35.9)	38 (14.3)	<0.001
Follow-up LGI endoscopy	207 (19.9)	34 (12.8)	0.010

Values are expressed as median (interquartile range) for continuous variables that did not show normal distribution, unless otherwise indicated. LGI, lower gastrointestinal; IBD, inflammatory bowel disease.

**Table 6:** Logistic regression analyses of factors associated with the conductance of a colonoscopy over a sigmoidoscopy.

	Univariate Analysis		Multivariate Analysis	
	OR (95% CI)	*p*-Value	OR (95% CI)	*p*-Value
Sex (male vs. female)	1.17 (0.89–1.53)	0.269		
Age at LGI endoscopy (years)	1.23 (1.20–1.26)	<0.001	1.19 (1.12–1.27)	<0.001
Weight at LGI endoscopy (kg)	1.06 (1.05–1.07)	<0.001	1.00 (0.99–1.02)	0.773
LGI endoscopy performance year (post- vs. pre-COVID-19)	1.77 (1.18–2.73)	0.008	1.60 (1.00–2.63)	0.058
Indication: LGI bleeding (yes vs. no)	0.27 (0.20–0.35)	<0.001	0.56 (0.37–0.83)	0.005
Indication: chronic diarrhea (yes vs. no)	3.20 (2.29–4.54)	<0.001	1.35 (0.84–2.15)	0.213
Diagnosis: IBD (yes vs. no)	3.36 (2.36–4.92)	<0.001	1.80 (1.20–2.77)	0.006

LGI, lower gastrointestinal; COVID-19, coronavirus infectious disease 2019; IBD, inflammatory bowel disease; OR, odds ratio; CI, confidence interval.

## Data Availability

All data generated in this study are available from the corresponding author upon request.
